# *Chromolaena odorata* (L.) R. M. King and H. Robinson Leaves Aqueous Extract Improves the Femoral Head in Ethanol-Induced Osteonecrosis in Rats

**DOI:** 10.1155/2023/5436771

**Published:** 2023-06-28

**Authors:** Florence Tsofack Ngueguim, Raceline Kamkumo Gounoue, Jean Hubert Donfack, Sandra Manefen Simo, Josiane Jouonzo, Rodrigue Ngapout Fifen, Paul Desire Djomeni Dzeufiet, Théophile Dimo

**Affiliations:** ^1^Department of Animal Biology and Physiology, Faculty of Science, University of Yaoundé 1, P.O. Box 812, Yaoundé, Cameroon; ^2^Department of Pharmaceutical Sciences, Faculty of Medicine and Pharmaceutical Sciences, University of Dschang, P.O. Box 96, Dschang, Cameroon

## Abstract

Chronic alcohol consumption damages bone formation and causes bone pathology, including osteonecrosis of the femoral head. The aim of this work was to evaluate the effects of the leaf aqueous extract of *Chromolaena odorata* (*C. odorata*) on the femoral head in ethanol-induced osteonecrosis in rats. Animals received alcohol (40°) at 3 g/kg for 12 weeks. A group of animals were sacrificed to attest to the instalment of osteonecrosis by using histopathological analysis. The remaining animals received alcohol concomitantly with the plant extract (150, 300, or 600 mg/kg) or diclofenac (1 mg/kg) for 28 additional days. At the end of the experimental period, biochemical parameters including total cholesterol, triglycerides, calcium, alkaline phosphatase (ALP), reduced glutathione (GSH), malondialdehyde (MDA), nitrite, superoxide dismutase (SOD), and catalase activities were measured. Histopathological and histomorphometry analyses of femurs were also assessed. The administration of alcohol, irrespective of the experimental period, induced a significant increase in total cholesterol (*p* < 0.05) and triglyceride (*p* < 0.01) and a decrease in ALP (*p* < 0.05) and calcium (*p* < 0.05–*p* < 0.001) levels. Intoxicated animals showed an alteration in oxidative stress parameters accompanied by a significant drop in bone cortical thickness and density with necrosis and marked bone resorption. The concomitant administration of the plant with ethanol reversed the alcohol-induced bone defect, characterized by the improvement of the lipid profile (*p* < 0.001), bone calcium concentration (*p* < 0.05), bone ALP activity (*p* < 0.001), oxidative stress parameters, improved cortical bone thickness (*p* < 0.01), and bone density (*p* < 0.05). These results are supported by the absence of bone resorption with an obvious effect at a dose of 300 mg/kg. The pharmacological effect of the extract on ethanol-induced osteonecrosis of the femoral head is probably due to its osteogenic, hypolipidemic, and antioxidant properties, justifying its use in Cameroonian folk medicine for articulation and bone pain management.

## 1. Introduction

Alcohol consumption has become one of the most serious substance abuse disorders worldwide [1]. Chronic alcohol consumption has a variety of harmful effects on several organs, including the brain, heart, liver, muscles, and bones [2]. Although recent studies have indicated that moderate levels of alcohol consumption may have beneficial effects on bone parameters and mineral density [3]. However, excessive alcohol consumption may be associated with impaired bone turnover and consequently bone fragility and a high risk of bone fracture [4]. Epidemiological investigations have shown that alcohol increases the risk of developing aseptic osteonecrosis of the femoral head [1]. Excessive alcohol intake has been associated with alterations in circulating lipids [5]. In fact, ethanol induces the differentiation of stromal cells in the bone marrow into adipocytes; likewise, it causes a significant increase in serum triglyceride and cholesterol levels. The deposition of triglyceride in osteocytes leads to pyknosis and an accumulated percentage of empty osteocytes, ultimately leading to osteocyte death [6]. The main purpose of the treatments used to manage this pathology is to reduce pain, prevent the progression towards the loss of sphericity of the femoral head, and give indications for conservative or prosthetic replacement surgeries.

*Chromolaena odorata (C. odorata)* is a plant belonging to the family of Asteraceae. The plant is empirically used for infective dermatitis [7]. Cameroonian trade practitioner and healers currently use the macerate of the plant for articulation and bone pain. In addition, pharmacological studies have revealed antimicrobial [8], anticancer [9], anticonvulsant [10], antidiarrhoeal [11], antifungal [12], antiplasmodial [13], antidiabetic [14], anti-inflammatory, antioxidant [15], and wound healing [16] properties. The present study was undertaken to examine the protective effect of the aqueous extract of *C. odorata* on ethanol-induced osteonecrosis of the femoral head.

## 2. Materials and Methods

### 2.1. Plant Material and Extraction Preparation

The leaves of *C. odorata* were harvested at Yaoundé III in the Center Region of Cameroon in May 2019. The plant was authenticated at the Cameroon National Herbarium in comparison with the specimen voucher N°8680/SRF/cam. The fresh leaves were cleaned, cut into pieces, and dried under a shade at room temperature. The decoction was carried out by boiling 600 g of fresh leaves in 3 L of tap water for 10 min following the traditional healer's instructions. The filtrate obtained was evaporated at 45°C in a drying cupboard to yield a 7.10% extract, which was kept at room temperature until use. The experimental doses used were obtained by measuring two cups of the filtrate extract according to the traditional healer's daily dose, dried, and quantified it corresponding to an average dose of 300 mg/kg. This dose was then flanked by the lower (150 mg/kg) and the higher (600 mg/kg) doses.

### 2.2. Animals

Six weeks old Wistar strain male rats weighing between 90 and 100 g were used in the present study. They were obtained from the animal house of the Faculty of Science at the University of Yaoundé I (Cameroon). Animals were given normal laboratory rat food and they received water *ad libitum*. The procedures followed the principles of laboratory animal use and care of the “European Community Guidelines (EEC Directive 2010/63/EEC) and were approved by the “Animal Ethical Committee” of the Faculty of Science, University of Yaoundé I.

### 2.3. Osteonecrosis Induction and Study Design

Animals received daily administration of ethanol (0.3 g/mL) at a dose of 3 g/kg for 12 weeks. A group of 5 animals (alcohol control group 1) were sacrificed to attest to the instalment of osteonecrosis. The remaining animals received ethanol and different treatments simultaneously as follows: alcohol control group 2 received ethanol and distilled water (10 mL/kg), one group received ethanol with diclofenac (1 mg/kg) as a reference drug, and three groups received ethanol with the plant extract at doses of 150, 300, and 600 mg/kg. All substances were administered by the oral route for twenty-eight days. At the end of the experimental period, all animals were sacrificed under anaesthesia using ketamine (30 mg/kg) and diazepam (10 mg/kg) via the intraperitoneal route. Arteriovenous blood was collected, and the serum was prepared for biochemical analysis (total cholesterol, triglyceride, and calcium) assessed using a commercial Kit (BIOLABO, France). Bones (femur) were carefully removed and weighted, and the volume and density were measured using the method of Oumarou et al. [17]. Bones' biochemical analysis was performed to determine the level of calcium and alkaline phosphatase (ALP) using a commercial kit from BIOLABO (France).

### 2.4. Assessment of the Aqueous Extract from *C. odorata* on Some Bone Parameters of Oxidative Stress

The femur was carefully removed, stripped of muscle tissue, and then weighted. The bones (0.2 g) were homogenised using 3 mL of phosphate buffer saline (pH, 7.4). The mixture was then centrifuged at 3000 rpm at 4°C for 30 min. The supernatant obtained was used to determine superoxide dismutase (SOD) activity using the method of Sinha [18], catalase activity according to the Misra and Fridovich protocol [19], reduced glutathione (GSH) concentration by the Ellman method [20], malondialdehyde (MDA), and nitrite concentrations according to Wilbur et al. [21] and Green et al. [22] procedures, respectively.

### 2.5. Histopathological Analysis

Histological analyses of the femurs were assessed by the hematoxylin-eosin staining method. The bones were conserved in 10% formalin and demineralized in a 10% HCl solution for 5 days. Isolated femur samples were embedded in paraffin, and sections with a thickness of 10 *μ*m were made using a microtome (Reichert-Jung 2030). Photographs of the sections were taken using a digital camera for the microscope (DCM, 35 : 350 K Pixels, USB 2.0) aided with appropriate filters. The cortical bone thickness was measured using Image J software version 1.49.

### 2.6. Statistical Analysis

Results are expressed as mean ± standard error mean. Statistical significance was determined by the one-way analysis of variance followed by the Tukey post-test using GraphPad Prism version 8.0.1 (GraphPad Software, San Diego, California, USA). Difference was considered significant if *p*  <  0.05.

## 3. Results

### 3.1. Effects of the *C. odorata* Leaves Aqueous Extract on Total Cholesterol and Triglyceride Concentrations in Alcohol-Intoxicated Rats

**Figure 1:**
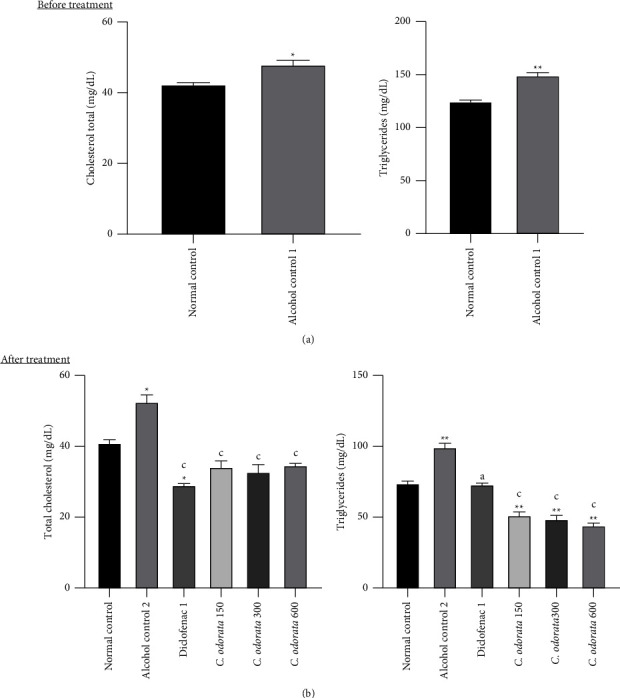
Effects of *C. odorata* leaf aqueous extract on total cholesterol and triglycerides in alcohol-intoxicated rat. Each bar represents the mean ± SEM (*n* = 5). ^*∗*^*p* <  0.05, ^*∗∗*^*p* <  0.01: significantly different compared to the normal control (1 or 2) group; ^a^*p* <  0.05, ^c^*p* <  0.001: significantly different compared to alcohol control (1 or 2) group. *C. odorata* 150, *C. odorata* 300, and *C. odorata* 600: rats treated concomitantly with the plant extract and alcohol at doses of 150, 300, and 600 mg/kg; Diclofenac1: rats treated concomitantly with alcohol and diclofenac at a dose of 1 mg/kg.

The effect of *C. odorata* on cholesterol and triglyceride concentrations is shown in Figure 1. The administration of alcohol provoked a significant increase in total cholesterol (*p* < 0.05) and triglyceride levels (*p* <  0.01) irrespective of the administration period (before and after treatment). The simultaneous administration of alcohol and the plant extract for 28 days significantly reduced the total cholesterol level (*p* <  0.01) by 34.85%, 51.05%, and 55.58% at respective doses of 150 mg/kg, 300 mg/kg, and 600 mg/kg compared to intoxicated animals. At the same doses, the plant extract also significantly dropped the triglyceride concentration, respectively, by 48.33% (*p* <  0.05), 35.76% (*p* <  0.01), and 36.36% (*p* <  0.01). The diclofenac used as an anti-inflammatory drug provoked a significant decrease (*p* < 0.001) in total cholesterol and triglyceride (*p* < 0.05) concentrations.

### 3.2. Effects of *C. odorata* Leaves Aqueous Extract on Serum and Bone Calcium in Alcohol-Intoxicated Rats

**Figure 2:**
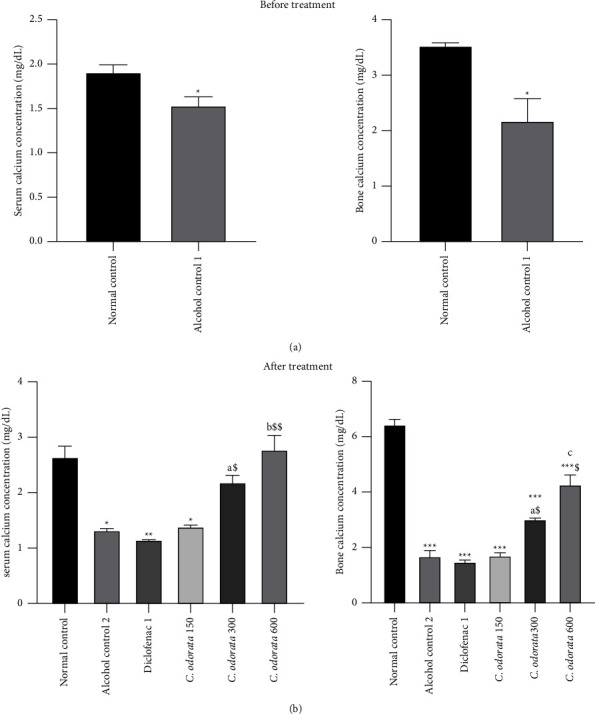
Effects of *C. odorata* leaves aqueous extract on serum and bone calcium. Each bar represents the mean ± SEM (*n* = 5). ^*∗*^*p* <  0.05, ^*∗∗*^*p*  <  0.01, ^*∗∗∗*^*p*  <  0.001: significantly different compared to the normal control group; ^a^*p* <  0.05, ^c^*p* <  0.001: significantly different compared to the alcohol control (1 or 2) group. ^$^*p* <  0.05, ^$$^*p*  <  0.01: significantly different compared to diclofenac, *C. odorata* 150, *C. odorata* 300, and *C. odorata* 600: rats treated concomitantly with plant extract and alcohol at the respective doses of 150, 300, and 600 mg/kg; Dicloenac1: rats treated concomitantly with alcohol and diclofenac at a dose of 1 mg/kg.

Alcohol treated-rats showed a significant decrease (*p* < 0.05) in serum and bone calcium concentrations by 19.89% and 38.24%, respectively, after three months of administration compared to normal rats (Figure 2(a)). Additional administration of alcohol for twenty-eight more days significantly emphasized the drop in serum calcium concentration by 50.00% (*p* < 0.05) and bone calcium by 73.91% (*p* < 0.001) as compared to their respective controls (Figure 2(b)). Concomitant administration of alcohol and plant extract resulted in an increase in serum calcium concentration by 65.90% (*p*  < 0.05) and 109.84% (*p*  < 0.01), bone calcium by 79.76% (*p*  < 0.05) and 154.16% (*p*  < 0.001) at respective doses of 300 and 600 mg/kg compared to their corresponding alcohol control groups. The plant extract at a dose of 150 mg/kg and diclofenac administered at 1 mg/kg failed to increase serum and bone calcium.

### 3.3. Effects of *C. odorata* Leaves Aqueous Extract on Bone ALP Activity

**Figure 3:**
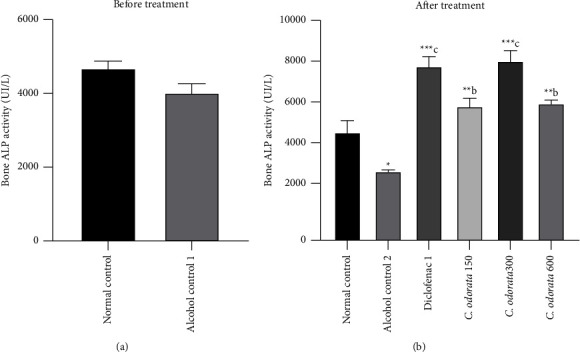
Effects of *C. odorata* leaves aqueous extract on bone ALP. Each bar represents the mean ± SEM (*n* = 5). ^*∗*^*p* <  0.05, ^*∗∗*^*p* <  0.01, and ^*∗∗∗*^*p*  <  0.001: significantly different compared to the normal control group; ^c^*p* <  0.001: significantly different compared to the alcohol control (1 or 2) group. *C. odorata* 150, *C. odorata* 300, and *C. odorata* 600: rats treated concomitantly with the plant extract and alcohol at doses of 150, 300, and 600 mg/kg; Diclofenac1: rats treated concomitantly with alcohol and diclofenac at a dose of 1 mg/kg.

The effect of *C. odorata* on ethanol-induced osteonecrosis is shown in Figure 3. The administration of alcohol for 12 weeks did not significantly decrease ALP activity; while a significant decrease in bone ALP activity was observed with the cumulative administration of alcohol for an additional 28 days as compared to the normal control. The plant extract caused a significant increase in bone ALP activity irrespective of the doses, with the highest effect at a dose of 300 mg/kg compared to the alcohol control group. The extract at all doses as well as diclofenac, caused a significant increase (*p* < 0.01 − *p* < 0.001) in bone ALP activity in comparison to the normal control.

### 3.4. Effects of *C. odorata* Leaves Aqueous Extract on NonEnzymatic Bone Oxidative Stress Parameters in Alcohol-Intoxicated Rats

**Figure 4:**
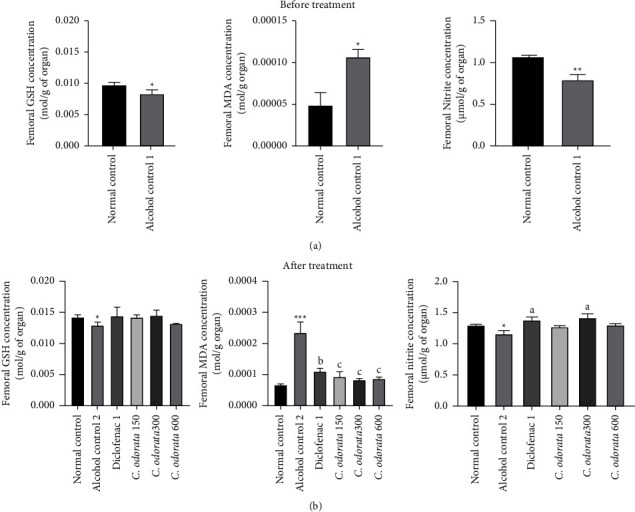
Effects of *C. odorata* leaves aqueous extracts on GSH, MDA and nitrite in alcohol-induced osteonecrosis. Each bar represents the mean ± SEM (*n* = 5). ^*∗*^*p* <  0.05, ^*∗∗*^*p*  <  0.01, and ^*∗∗∗*^*p*  <  0.001: significantly different compared to the normal control group; ^a^*p* <  0.05, ^c^*p* <  0.001: significantly different compared to the alcohol control (1 or 2) group. *C. odorata* 150, *C. odorata* 300, and *C. odorata* 600: rats treated concomitantly with plant extract and alcohol at doses of 150, 300, and 600 mg/kg; Diclofenac1: rats treated concomitantly with alcohol and diclofenac at a dose of 1 mg/kg.

The alcohol supplementation induced a significant decrease in glutathione (*p* <  0.05) and nitrite (*p* < 0.01) concentrations, whereas a significant increase was observed in MDA (*p* < 0.05) concentrations regardless of the administration time (Figures 4(a) and 4(b)). Alcohol consumption for an additional 28 days also significantly increased the MDA concentration by 71.77% (*p* < 0.001). In comparison to the alcohol group, daily administration of the plant extract inhibited the decrease in GSH concentration, though nonsignificant (150 mg/kg and 300 mg/kg). Simultaneous administration of ethanol and the extract at doses of 150 mg/kg, 300 mg/kg, and 600 mg/kg resulted in a significant decrease (*p*  < 0.001) in MDA concentration respectively by 60.16%, 65.25%, and 63.38%. The nitrite content was significantly increased (*p* < 0.05), with a marked effect at a dose of 300 mg/kg compared to the ethanol group. Diclofenac administered under the same conditions as the plant extract caused a significant decrease (*p* < 0.01) in MDA and an increase (*p* < 0.05) in nitrite concentration as compared to the alcohol control.

### 3.5. Effects of *C. odorata* Leaves Aqueous Extract on Enzymatic Bone Oxidative Stress Parameters in Alcohol-Intoxicated Rats

**Figure 5:**
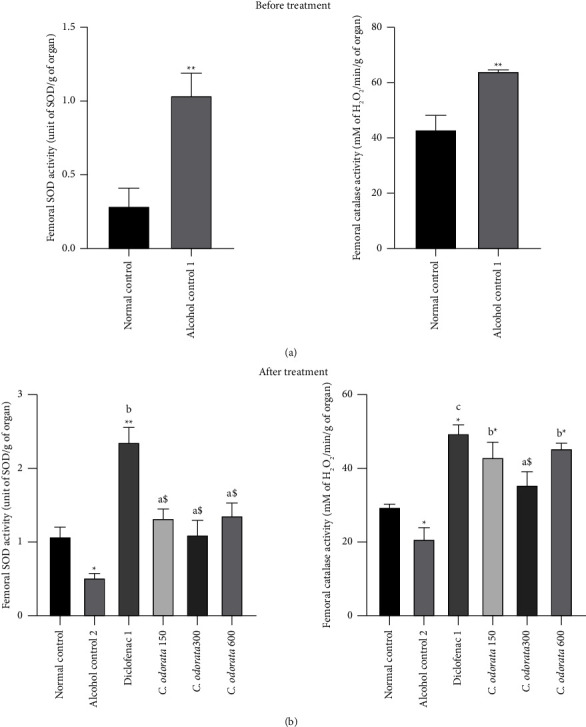
Effects of *C. odorata* leaves aqueous extract on SOD and catalase activities. Each bar represents mean ± SEM (*n* = 5). ^*∗*^*p* <  0.05, ^*∗∗*^*p*  <  0.01, and ^*∗∗∗*^*p*  <  0.001: significantly different compared to the normal control group; ^a^*p* <  0.05, ^c^*p* <  0.001: significantly different compared to the alcohol control (1 or 2) group. ^$^*p* <  0.05: significantly different compared to diclofenac, *C. odorata* 150, *C. odorata* 300, and *C. odorata* 600: rats treated concomitantly with the plant extract and alcohol at doses of 150, 300, and 600 mg/kg; Diclo1: rats treated concomitantly with alcohol and diclofenac at a dose of 1 mg/kg.

The administration of alcohol at a dose of 3 g/kg for three months resulted in a significant increase (*p* < 0.001) in the SOD and catalase activities before the treatment (Figure 5(a)). Whereas, the supplementation of alcohol for an additional 28 days induced a significant decrease (*p* < 0.05) of SOD and catalase activities compared to the normal control (Figure 5(b)). The administration of the plant extract for 28 days significantly reversed the deleterious effect of alcohol on SOD (*p* < 0.05) and catalase (*p* < 0.001) activities at doses of 150 mg/kg and 600 mg/kg. At a dose of 150 mg/kg, the extract also improved (*p* < 0.05) SOD and catalase activities as compared to the alcohol group; and interestingly, the values were close to those of normal rats.

### 3.6. Effects of *C. odorata* Leaves Aqueous Extract on the Cortical Bone Thickness and Bone Density of Alcohol-Intoxicated Rats

**Figure 6:**
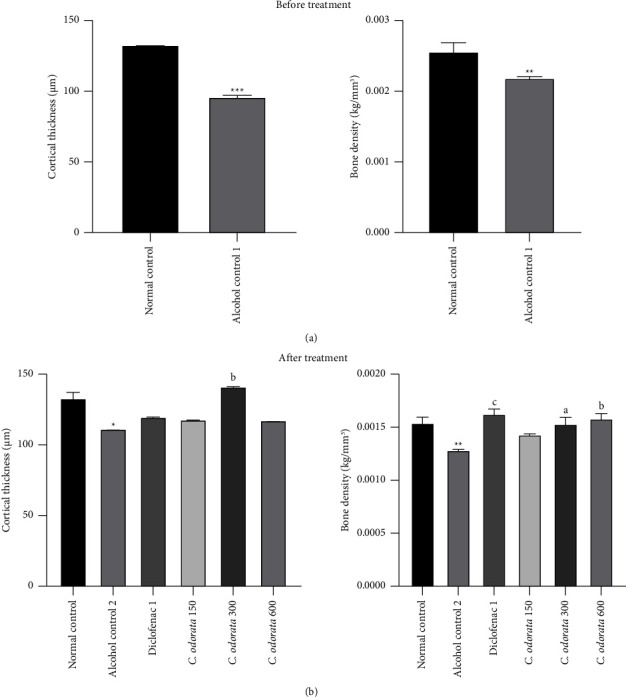
Effects of *C. odorata* leaves aqueous extract on bone cortical thickness and density. Each bar represents mean ± SEM (*n* = 5). ^*∗*^*p* <  0.05, ^*∗∗*^*p* <  0.01, and ^*∗∗∗*^*p* <  0.001: significantly different compared to the normal control group; ^a^*p* <  0.05, ^c^*p* <  0.001: significantly different compared to the alcohol control (1 or 2) group. *C. odorata* 150, *C. odorata* 300, and *C. odorata* 600: rats treated concomitantly with the plant extract and alcohol at doses of 150, 300, and 600 mg/kg; Diclo1: rats treated concomitantly with alcohol and diclofenac at a dose of 1 mg/kg.

The effect of the aqueous extract of *C. odorata* leaves on bone cortical thickness and bone density is shown in Figure 6. The oral administration of alcohol for three months induced a significant decrease in bone cortical thickness (*p* < 0.001) and bone density (*p* < 0.01) as compared to normal control rats. Concomitant administration of the plant extract with alcohol for more than 28 days significantly increased cortical thickness (*p* < 0.01) and bone density (*p* < 0.05) at a dose of 300 mg/kg in comparison with the alcohol control (Figures 6(a) and 6(b)). The plant extract failed to improve cortical thickness at doses of 150 and 600 mg/kg but significantly increased bone density at a dose of 600 mg/kg (*p* < 0.01) as compared to the ethanol control group.

### 3.7. Effects of *C. odorata* Leaves Aqueous Extract on the Femoral Head of Alcohol-Intoxicated Rats

**Figure 7:**
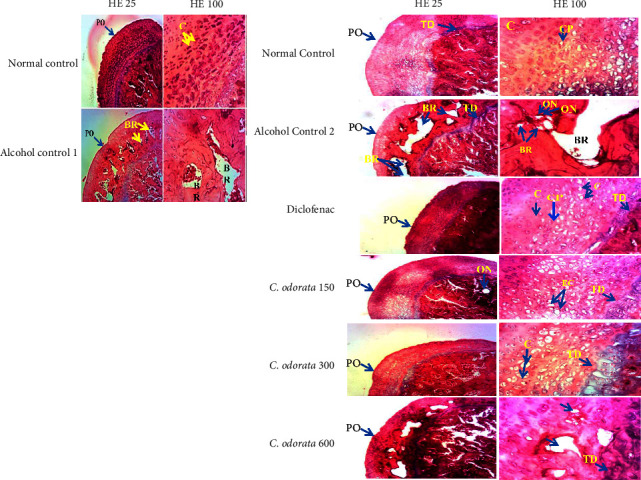
Microphotographs of the femoral head (HE 25x and HE 100x) in ethanol-induced osteonecrosis. *C. odorata* 150, *C. odorata* 300, and *C. odorata* 600: rats treated concomitantly with the plant extract and alcohol at doses of 150, 300, and 600 mg/kg; Diclo1: rats treated concomitantly with alcohol and diclofenac at a dose of 1 mg/kg. BR: bone resorption, PO: perichondrium, C: chondrocyte, EC: empty chondroplast, CP: chondroplast, ON: osteonecrosis TD: tidemark, normal control: rats receiving distilled water, alcohol control 1: rats receiving ethanol 40° (3 g/kg) for 12 weeks.

Figure 7 shows the effect of the extract on the femoral head of alcohol-intoxicated rats. The femoral head section of normal control shows a distinct perichondrium and Tidemark with a normal aspect of chondrocytes (normal control). The administration of alcohol provoked bone resorption which was accentuated when alcohol administration was prolonged for 28 additional days (alcohol control 1 group). In addition, there was marked necrosis in the alcohol treated group (alcohol control 2 group). The plant extract prevented the instalment of bone damage with a marked effect at a dose of 300 mg/kg (C. *odorata* 150–300 mg/kg). The highest dose (600 mg/kg) failed to protect the bone against alcohol-induced bone resorption.

## 4. Discussion

The present study was carried out to investigate the effects of C*. odorata* aqueous extract on ethanol-induced osteonecrosis of the femoral head of a rat. In the present study, the administration of ethanol (40°) at a dose of 3 g/kg irrespective of the period induced a significant decrease in bone mineral density. These results suggest that ethanol has harmful effects on bone metabolism. Indeed, alcohol slows down the bone remodeling process [23] by several mechanisms: the increase in sclerostin serum (an inhibitor of bone formation), which in turn increases osteoblast apoptosis through activation of caspase pathways [24], the stimulation of osteoclastogenesis by increasing the RANKL/OPG (receptor activator of NF-*κ*B ligand-osteoprotegerin) ratio [25, 26]. In addition, ethanol induces the differentiation of mesenchymal cells into adipocytes [27, 28], which increase adipose cells within the bone marrow contributing to the decrease in bone mass. The administration of the extract at different doses increased bone density, suggesting that *C. odorata* aqueous extract could inhibit ethanol-induced necrosis and/or stimulate the bone formation process. ALP is a marker of bone formation secreted by osteoblasts and playing a role in bone mineralization [29]. The ethanol administration for 12 weeks led to a significant decrease in serum and bone ALP, attesting once more to the negative impact of ethanol on the bone formation process [30]. Indeed, ethanol inhibits the differentiation of mesenchymal stem cells into osteoblasts [6] by suppressing the Wnt/*β*-Catenin signaling pathway [31], thus promoting the differentiation of these pluripotent cells into adipocytes [32]. The results obtained in this study confirm the effects of alcohol on the levels of ALP and bone mineralization. The administration of the extract of *C. odorata* led to an increase in the concentration of bone ALP at different doses, indicating that the extract would have lifted the inhibitory effect of ethanol on the transformation of mesenchymal stem cells into osteoblasts. Calcium and phosphorus are necessary building blocks for bone formation and are stored in the bone as hydroxyapatite, which ensures bone rigidity. As observed in the present study, administration of ethanol significantly decreased serum and bone calcium levels. In fact, excessive alcohol consumption has been demonstrated as a cause of malnutrition [33] expressing by harmful effects on the absorption processes, metabolism, and excretion of micronutrients [23, 34], thereby increasing the risk of the occurrence of osteonecrosis. Thus, in this context, the vitamin D deficiency caused by ethanol induces a decrease in the intestinal reabsorption of calcium, hence its negative impact on bone metabolism. Administration of the extract at the gradual doses of 300 mg/kg and 600 mg/kg resulted in a significant increase in serum and bone calcium, testifying its ability to promote bone mineralization. This result would be explained not only by the capacity of the extract to reduce the deleterious effects of ethanol on the metabolism of micronutrients but also by the presence of minerals in the plant extract such as calcium, phosphorus, magnesium and, sodium which play a role in the process of bone mineralization [35–37]. Oxidative stress is known as one of the mechanisms involved in osteonecrosis [38, 39]. In the femur, MDA concentration increased while catalase SOD activity and GSH level decreased, expressing the establishment of oxidative stress in ethanol-induced osteonecrosis. It is well demonstrated that ethanol increases the activity of the enzyme NADPH oxidase (NOX), thus leading to an increase in the production of free radicals within osteoblasts and the expression of RANKL, which activates osteoclastogenesis [40]. Ethanol-induced lipoperoxidation contributes to cytomembrane lesions; degeneration of the arterioles, and arteriosclerosis, which lead to ischemia in the target organ. In fact, lipid peroxidation induced by alcohol and its metabolites also leads to worsening of the ischemic state of osteocytes and therefore leads to osteonecrosis [41]. The decrease in nitrite levels could result from the disruption of the release of nitric oxide (NO). Indeed, ethanol could have acted by stimulating modulators of apoptosis, such as inducible nitric oxide synthase (iNOS), leading to locally toxic levels of NO in osteoblasts and osteocytes, followed by cell death and consequently the development of osteonecrosis of the femoral head [42]. The treatment of animals with the extract at doses of 150 and 300 mg/kg has resulted in the correction of these parameters indicating the antioxidant activity of the plant extract. The different osteogenic and antioxidant properties demonstrated by the aqueous extract of *C. odorata* could be due to secondary metabolites within the plant. In fact, phytochemical screening of this extract revealed the presence of tannin, saponin, flavonoids, phenols, and coumarins [43, 44]. Most of these biometabolites, such as flavonoid [45, 46] and saponins [47], promote bone regeneration, while coumarins increase bone morphogenic protein-2 expression and thus enhance bone formation [48]. Moreover, these secondary metabolites particularly flavonoids, and phenols [49, 50] are classes of compounds recognized for their ability to trap ROSs. The overall biological activities of the extract may contribute to improve bone regeneration in the plant extract-treated-rats. These biochemical results were strengthened by histological sections showing degenerated chondrocytes in the femoral epiphysis and the presence of empty lacunae testifying bone resorption in animals receiving ethanol [41]. The absence of lacunae in animals receiving the extract suggests the inhibitory activity of the extract against the deleterious impact of ethanol on the femoral head, with marked activity in animals receiving the extract at a dose of 300 mg/kg. Furthermore, the reduction in cortical bone thickness in animals receiving ethanol demonstrates its negative impact on the process of bone metabolism by stimulating bone degeneration, justifying the reduction in bone density in these animals. The administration of the extract induced an improvement of these parameters suggesting the capacity of the extract to counteract the effects of ethanol on the bone damage by lifting the inhibitory effect of ethanol on the differentiation of stem cells into the osteoblastic line.

## 5. Conclusion

The concomitant administration of the plant extract with ethanol reversed alcohol-induced bone defects, characterized by the improvement of the lipid profile, bone calcium concentration, and bone ALP activity. The extract also reduced oxidative stress parameters increased cortical bone thickness and bone density. The extract protected the bone against resorption induced by alcohol, with a pronounced effect at a dose of 300 mg/kg. These results confirm the use of this plant extract for the management of articulation and bone pain in Cameroonian folk medicine. Supplementary studies are needed to determine the mechanism of action of the *C. odorata* aqueous extract-induced-osteoprotective effect.

## Data Availability

The data used to support the findings of this study are available from the corresponding author upon request.

## Abbreviations

ALP: Alkaline phosphatase
GSH: Reduced glutathione
MDA: Malondialdehyde
iNOS: Inducible nitric oxide synthase
NO: Nitric oxide
SOD: Superoxide dismutase
RANKL/OPG: Receptor activator of NF-*κ*B ligand-Osteoprotegerin
ROS: Reactive oxygen species.
